# HIF-1 and c-Src Mediate Increased Glucose Uptake Induced by Endothelin-1 and Connexin43 in Astrocytes

**DOI:** 10.1371/journal.pone.0032448

**Published:** 2012-02-23

**Authors:** José Carlos Valle-Casuso, Ana González-Sánchez, José M. Medina, Arantxa Tabernero

**Affiliations:** Departamento de Bioquímica y Biología Molecular, Instituto de Neurociencias de Castilla y León (INCYL), Universidad de Salamanca, Salamanca, Spain; Instituto de Investigación Sanitaria INCLIVA, Spain

## Abstract

In previous work we showed that endothelin-1 (ET-1) increases the rate of glucose uptake in astrocytes, an important aspect of brain function since glucose taken up by astrocytes is used to supply the neurons with metabolic substrates. In the present work we sought to identify the signalling pathway responsible for this process in primary culture of rat astrocytes. Our results show that ET-1 promoted an increase in the transcription factor hypoxia-inducible factor-1α (HIF-1α) in astrocytes, as shown in other cell types. Furthermore, HIF-1α-siRNA experiments revealed that HIF-1α participates in the effects of ET-1 on glucose uptake and on the expression of GLUT-1, GLUT-3, type I and type II hexokinase. We previously reported that these effects of ET-1 are mediated by connexin43 (Cx43), the major gap junction protein in astrocytes. Indeed, our results show that silencing Cx43 increased HIF-1α and reduced the effect of ET-1 on HIF-1α, indicating that the effect of ET-1 on HIF-1α is mediated by Cx43. The activity of oncogenes such as c-Src can up-regulate HIF-1α. Since Cx43 interacts with c-Src, we investigated the participation of c-Src in this pathway. Interestingly, both the treatment with ET-1 and with Cx43-siRNA increased c-Src activity. In addition, when c-Src activity was inhibited neither ET-1 nor silencing Cx43 were able to up-regulate HIF-1α. In conclusion, our results suggest that ET-1 by down-regulating Cx43 activates c-Src, which in turn increases HIF-1α leading to the up-regulation of the machinery required to take up glucose in astrocytes. Cx43 expression can be reduced in response not only to ET-1 but also to various physiological and pathological stimuli. This study contributes to the identification of the signalling pathway evoked after Cx43 down-regulation that results in increased glucose uptake in astrocytes. Interestingly, this is the first evidence linking Cx43 to HIF-1, which is a master regulator of glucose metabolism.

## Introduction

Endothelins (ET-1, ET-2 and ET-3) are vasoactive peptides found in many tissues [1]. The first member of this family, ET-1 is present in brain endothelial cells [2], neurons [3], and astrocytes [4], and its secretion increases in several pathologies, such as cerebral ischemia [5], Alzheimer disease [6], HIV infection [7], [8], reactive gliosis [4], [9], [10] and astrocytic tumours [11]. In astrocytes, ET-1 behaves as a growth factor, exerting important biological effects such as changes in protein content and morphology [12], the induction of proliferation [13], [14], [15], [16] and the increase in the rate of glucose uptake [16], [17], [18].

The regulation of glucose uptake in astrocytes is an important aspect of brain function since glucose taken up by astrocytes is used not only by astrocytes but also to supply the neurons with metabolic substrates required to sustain synaptic transmission [19], [20]. Astrocytes are connected through gap junctions [21], composed mainly of connexin43 (Cx43) [22]. This intercellular communication provides the basis for several important astrocytic functions [23]. For instance, gap junction channels allow the passage from cell to cell of glucose and other metabolites, contributing to the distribution of metabolic substrates from blood to different regions [17], [20], [23]. Various physiological and pathological signals promote changes in gap junctional communication and Cx43 expression (for a review, see [23]). One of these signals is ET-1, which rapidly inhibits gap junctional communication between astrocytes and after long-term exposure (24 h) causes the down-regulation of Cx43 [24], [25]. In fact, we have shown that the down-regulation of Cx43 exerted by ET-1 is involved in the increase in the rate of glucose uptake observed in astrocytes [26]. This effect includes the up-regulation of the glucose transporter GLUT-1 and the induction of GLUT-3, an isoform not normally expressed in astrocytes [18], [26]. Intracellular glucose is quickly phosphorylated by hexokinase (Hx) to glucose-6-phosphate, which is a charged molecule that cannot pass back through the plasma membrane and becomes trapped within the cell. Both type I (Hx-1) and type II (Hx-2) hexokinase are up-regulated by ET-1 in astrocytes [18], [26].

Hypoxia-Inducible Factor (HIF)-1α/β heterodimer is a master transcription factor for several genes involved in glucose uptake, angiogenesis, glycolysis, pH balance and metastasis. Among the genes activated by HIF-1 are GLUT-1, GLUT-3, Hx-1 and Hx-2 (revised in [27]). While HIF-1β is stable and constitutively expressed, HIF-1α is highly regulated, as well as susceptible to oxygen-dependent degradation due to the sequential action of oxygen-dependent prolyl hydroxylases and the VHL ubiquitin ligase. Therefore, under hypoxic conditions HIF-1α is stabilized, dimerizes with HIF-1ß and activates target genes. It should be mentioned that although HIF is mainly activated under hypoxia, several factors activate HIF-1α under normoxic conditions. Intriguingly, endothelins are among the factors with the ability to activate HIF-1α under normoxic conditions in melanoma and ovarian carcinoma cells [28], [29], [30]. Furthermore, oncogenes, such as c-Src prevent hydroxylation-dependent ubiquitinylation of HIF-1α, thus stabilizing it under normoxic conditions [31], [32], [33].

The intracellular carboxyl tail of Cx43 interacts with a large number of signalling and scaffolding proteins [34], [35], thereby regulating cell functions such as cell adhesion, migration and proliferation [36], [37], [38], [39]. One of these interacting proteins is the non-receptor tyrosine kinase c-Src [40], [41], [42]. Interestingly, we have recently shown that the interaction between Cx43 and c-Src promotes the inactivation of the oncogenic activity of c-Src [43]. Although it is well described that ET-1 increases the rate of glucose uptake by a Cx43-dependent mechanism that includes the up-regulation of GLUT-1, GLUT-3, Hx-1 and Hx-2 [18], [26], so far there have been no studies examining the mechanism by which Cx43 regulated these genes. Since GLUT-1, GLUT-3, Hx-1 and Hx-2 are targets of the transcription factor HIF-1α, and this factor can be activated by c-Src, which interacts with Cx43, in this work we aimed to investigate the participation of HIF-1α and c-Src on ET-1 modulation of the rate of glucose uptake in astrocytes.

## Results

### ET-1 up-regulates HIF-1α by decreasing Cx43 expression in astrocytes

**Figure 1:**
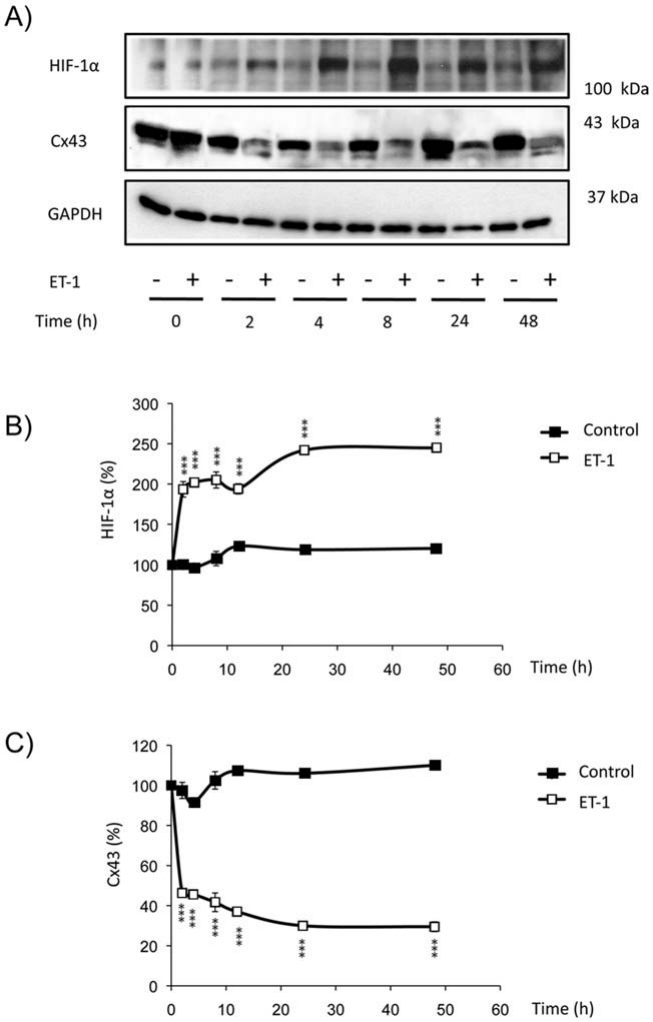
Effect of ET-1 on the expression of HIF-1α in astrocytes. Astrocytes were incubated in the absence (control) or presence of 0.1 µM ET-1 for the indicated times. Then, the expression of HIF-1α and Cx43 was analysed by Western blot. **A**) Representative Western blot of HIF-1α, Cx43 and GAPDH showing that ET-1 up-regulated HIF-1α and down-regulated Cx43. **B**) HIF-1α quantification. **C**) Cx43 quantification. The results are expressed as percentages of the level found in the controls at time 0. ***p<0.001 versus the corresponding controls.

In previous work we showed that ET-1 increased the rate of glucose uptake in astrocytes. Thus, ET-1 up-regulated GLUT-1 and Hx-1 and induced the expression of isoforms not normally expressed in astrocytes, such as GLUT-3 and Hx-2 [18], [26]. Since these proteins are target genes of HIF-1α, in this work we investigated whether the treatment with ET-1 modified HIF-1α levels in astrocytes, as it has been shown in other cell types [28], [29], [30]. Our results show that the treatment with 0.1 µM ET-1 strongly increased (by about 150%) the levels of HIF-1α (Figures 1A and B). This effect was evident after two hours of treatment and persisted for at least 48 h.

**Figure 2:**
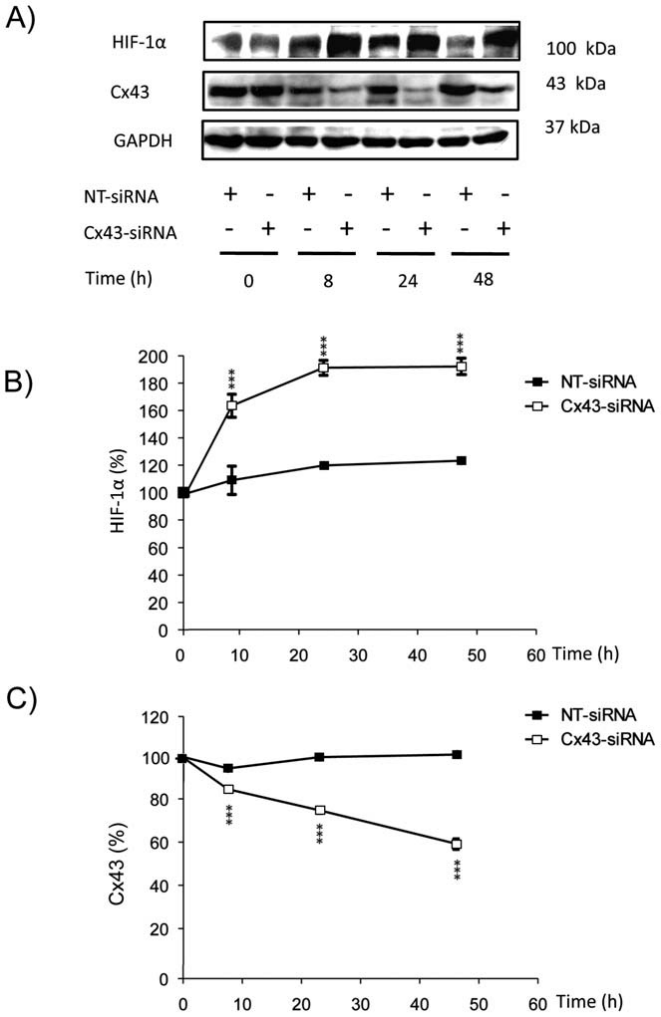
Effect of silencing Cx43 on the expression of HIF-1α in astrocytes. Astrocytes were transfected with NT-siRNA or with Cx43-siRNA. At the indicated times the expression of HIF-1α and Cx43 was analysed by Western blot. **A**) Representative Western blot of HIF-1α, Cx43 and GAPDH showing that the decrease in Cx43 expression was concomitant with HIF-1α up-regulation. **B**) HIF-1α quantification. **C**) Cx43 quantification. The results are expressed as percentages of the level found in the NT-siRNA condition at time 0. ***p<0.001 versus the corresponding NT-siRNA values.

**Figure 3:**
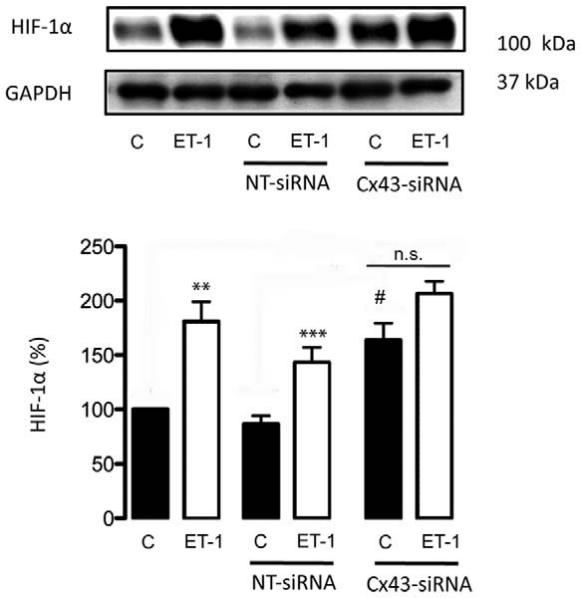
Effect of ET-1 on the expression of HIF-1α in Cx43-silenced astrocytes. Astrocytes were transfected with NT-siRNA or with Cx43-siRNA as indicated. After 48 h, cells were incubated in the absence (control, C) or presence of 0.1 µM ET-1 for 24 h. HIF-1α levels were analysed by Western blot. Note that the differences between ET-1 and Control in Cx43-silenced astrocytes were lower than those found between ET-1 and Control in NT-siRNA or in non-transfected astrocytes. The results are expressed as percentages of the level found in control non-transfected cells. ***p<0.001 versus the corresponding controls; ^#^p<0.05 versus the corresponding non-transfected cells. n.s. not significant.

The inhibition of gap junctional communication promoted by ET-1 in astrocytes is well documented [12], [16], [17], [44], [45], [46], [47]. Thus, ET-1 triggers very fast changes (within 3–10 minutes) in the gap junctional communication and in the phosphorylation status of Cx43 [48]. Changes in gap junctional communication and in the phosphorylation status of Cx43 are associated with changes in Cx43 interaction with other proteins and with Cx43 endocytosis [41], [42], [48]. Thus, a prolonged (24 hours) exposure to ET-1 reduces Cx43 expression in astrocytes [24], [25]. Interestingly, the down-regulation of Cx43 promoted by ET-1 coincided with the up-regulation of HIF-1α (Figure 1C). Since our previous work indicated that the effect of ET-1 on glucose uptake was due to the reduction in Cx43 expression, we investigated the effect of decreasing Cx43 expression on HIF-1α levels. Thus, by using specific siRNA against Cx43 [26], [43] we found that 48 h after the transfection with Cx43-siRNA the level of Cx43 was reduced by about 50% (Figures 2A and 2C) and the expression of HIF-1α was increased by about 80% (Figure 2B), when compared to astrocytes transfected with a non-targeting siRNA (NT-siRNA). Consequently, our results show that silencing Cx43 up-regulated HIF-1α. In addition, Figure 3 shows that the difference in HIF-1α levels between ET-1 and the control was not statistically significant in cells transfected with Cx43-siRNA, indicating that ET-1 was not able to further increase significantly the levels of HIF-1α in Cx43-silenced astrocytes.

### HIF-1α mediates the increase in glucose uptake promoted by ET-1

**Figure 4:**
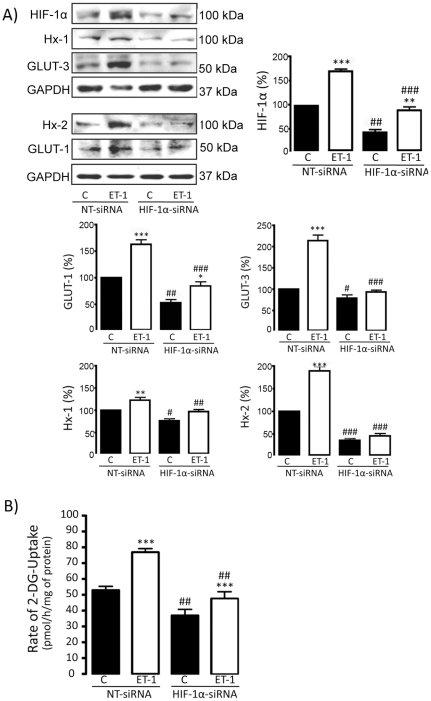
Effect of ET-1 on glucose uptake in HIF-1α-silenced astrocytes. Astrocytes were transfected with NT-siRNA or with HIF-1α-siRNA. After 48 h, astrocytes were incubated in the absence (control, C) or presence of 0.1 µM ET-1 for 24 h. **A**) HIF-1α, Hx-1, GLUT-3, Hx-2, GLUT-1 and GAPDH Western blots and quantification. The results are the means ± SEM of at least three independent experiments and they are expressed as percentages of the level found in the control NT-siRNA. **B**) Glucose uptake expressed as pmol of 2-deoxyglucose (2-DG) taken up per hour and per milligram of protein. The results show that the down-regulation of HIF-1α levels promoted by HIF-1α-siRNA decreased the rate of glucose uptake and the expression of GLUT-1, GLUT-3, Hx-1 and Hx-2, both in the control and in the ET-1 treated astrocytes. ***p<0.001, **p<0.01 and *p<0.05 versus the corresponding controls (C); ^###^p<0.001, ^##^p<0.01 and ^#^p<0.05 versus the corresponding NT-siRNA.

Since our results indicate that the axis ET-1/Cx43 up-regulates HIF-1α, which is a transcription factor involved in the regulation of glucose uptake, our next goal was to investigate the participation of HIF-1α in the increase in the rate of glucose uptake promoted by ET-1 in astrocytes. To do so, the expression of HIF-1α was down-regulated by 3 different and specific siRNAs against HIF-1α (HIF-1α-siRNA) and the results compared with those obtained with NT-siRNA. Our results show that 48 h after the transfection with HIF-1α-siRNA the expression of HIF-1α was reduced by about 50% when compared with NT-siRNA (Figure 4A) and this effect persisted for at least 72 h (Figure S1). Next, the effect of ET-1 was tested in astrocytes transfected with NT-siRNA or with HIF-1α-siRNA for 48 h. Our results show that the up-regulation of HIF-1α promoted by ET-1 in astrocytes transfected with NT-siRNA was reduced in HIF-1α-silenced astrocytes (Figure 4A). In a similar way, silencing HIF-1α strongly reduced the up-regulation of GLUT-1, GLUT-3, Hx-1 and Hx-2 promoted by ET-1 in astrocytes transfected with NT-siRNA (Figure 4A). Importantly, this effect was also observed when the rate of glucose uptake was analyzed. Thus, the rate of glucose uptake was reduced by HIF-1α-siRNA both in the control and in the ET-1 treated astrocytes (Figure 4B), suggesting that HIF-1α participates in the effect of ET-1 on glucose uptake.

### c-Src mediates the increase in glucose uptake promoted by ET-1

**Figure 5:**
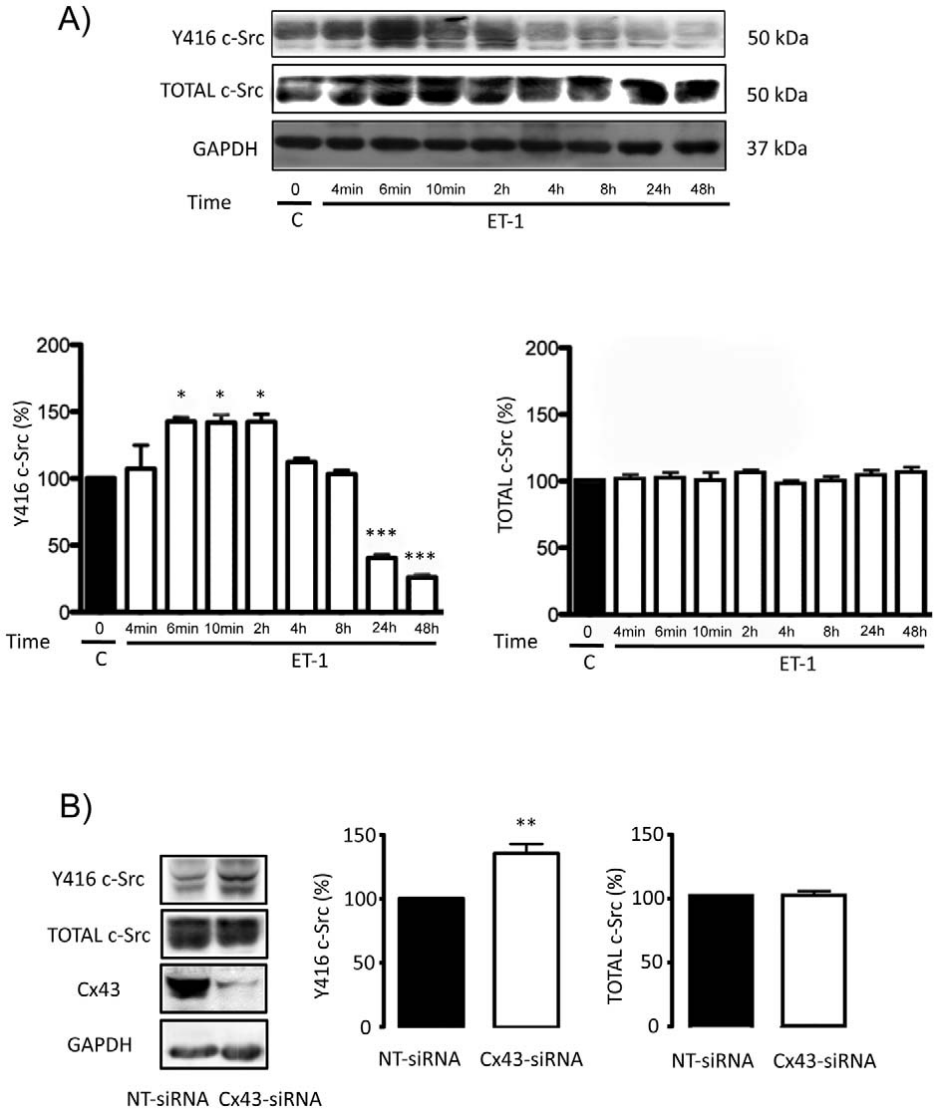
Effect of ET-1 and Cx43 on c-Src activity in astrocytes. **A**) Astrocytes were incubated in the absence (control, C) or presence of 0.1 µM ET-1 for the indicated times, proteins were extracted and Y416 c-Src and total c-Src were analysed by Western blot. The results are expressed as percentages of the level found in the control and they show that ET-1 rapidly and transiently increased Y416 c-Src without affecting significantly total c-Src. ***p<0.001; *p<0.05 versus control. **B**) Astrocytes were transfected with NT-siRNA or with Cx43-siRNA. After 48 h, proteins were extracted and Y416 c-Src, total c-Src and Cx43 were analysed by Western blot. The results are expressed as percentages of the level found in the control and they show that silencing Cx43 increased Y416 c-Src without affecting significantly total c-Src. **p<0.01 versus NT-siRNA.

Our recent work shows a direct relationship between Cx43 and c-Src activity [43]. Since c-Src has been shown to up-regulate HIF-1α [31], [32], [33], we investigated whether c-Src mediates the effect of ET-1 and Cx43 on glucose uptake. To measure c-Src activity we analysed the levels of c-Src phosphorylated at Tyr-416 (Y416 c-Src), the active form of this tyrosine kinase [49], [50]. Our results show that ET-1 transiently increased c-Src activity. Thus, ET-1 rapidly increased Y416 c-Src (within 6 minutes) and this effect was maintained for two hours, decreasing thereafter (Figure 5A). Meanwhile, the total amount of c-Src remained constant. Interestingly, silencing Cx43 also increased c-Src activity without changing the amount of total c-Src (Figure 5B).

**Figure 6:**
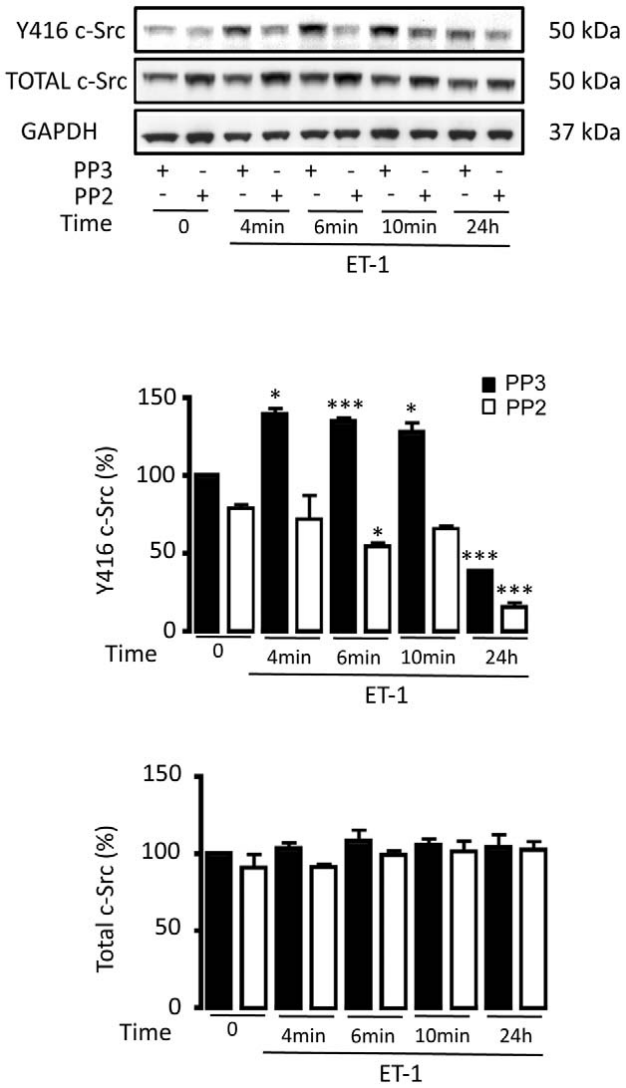
Inhibition of c-Src activity in astrocytes by PP2. Astrocytes were preincubated with 100 ng/µL PP2 (c-Src inhibitor) or 100 ng/µL PP3 (inactive analogue) for 1 h and these agents were maintained for the rest of the experiment. Then, cells were incubated in the absence or presence of 0.1 µM ET-1 for the indicated times and proteins were extracted for the analysis of Y416 c-Src and total c-Src by Western blot. The results are expressed as percentages of the level found in the control (PP3, time 0) and they show that PP2 inhibited the activation of c-Src (Y416 c-Src) promoted by ET-1. ***p<0.001; *p<0.05 versus the corresponding time 0.

**Figure 7:**
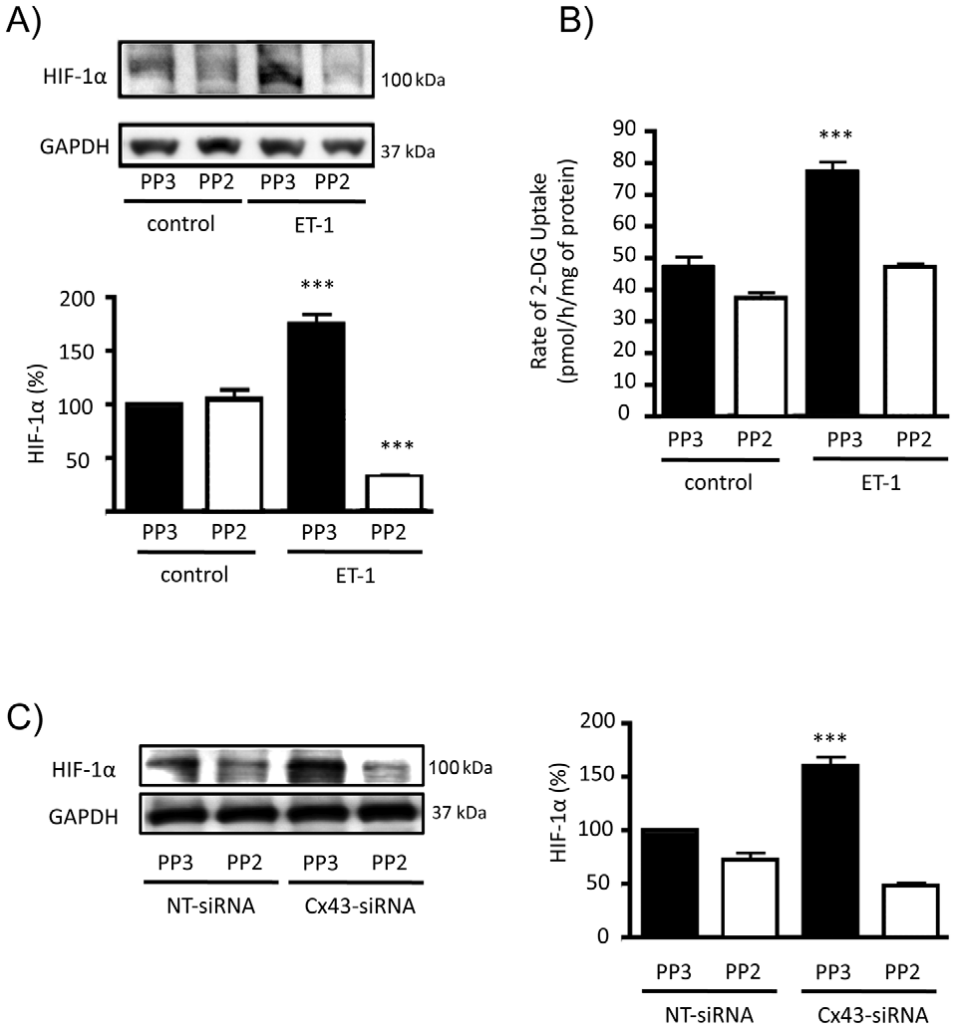
Effect of ET-1 and Cx43 on HIF-1α expression and glucose uptake when c-Src is inhibited. Astrocytes were preincubated with 100 ng/µL PP2 (c-Src inhibitor) or 100 ng/µL PP3 (inactive analogue) for 1 h. Then, cells were incubated in the absence (control) or presence of 0.1 µM ET-1 for 24 h. **A**) HIF-1α Western blot and quantification. The results are expressed as percentages of the level found in the controls treated with PP3 and they show that the inhibitor of c-Src PP2 prevented the up-regulation of HIF-1α promoted by ET-1. ***p<0.001 versus the absence of ET-1. **B**) Glucose uptake expressed as pmol of 2-deoxyglucose taken up per hour and per milligram of protein. The results show that the inhibitor of c-Src PP2 prevented the increase in the rate of glucose uptake promoted by ET-1. ***p<0.001 versus the absence of ET-1. **C**) Astrocytes were preincubated with 100 ng/µL PP2 or 100 ng/µL PP3 for 1 h. Then, cells were transfected with NT-siRNA or with Cx43-siRNA and after 48 h HIF-1α was analysed by Western blot. The results are expressed as percentages of the level found in the PP3 NT-siRNA and they show that the inhibitor of c-Src PP2 prevented the up-regulation of HIF-1α promoted by silencing Cx43. ***p<0.001 versus the corresponding NT-siRNA.

In order to investigate whether the activation of c-Src was responsible for HIF-1α up-regulation, the activity of c-Src was inhibited by PP2 and the inactive analogue PP3 was used as a control. Thus, while in the presence of PP3, ET-1 immediately increased c-Src activity, in the presence of PP2 the effect of ET-1 on c-Src was abrogated (Fig. 6). Next, the effect of ET-1 and Cx43 on HIF-1α was tested when c-Src was inhibited by PP2. Our results show that ET-1 was not able to up-regulate HIF-1α (Figure 7A) when c-Src was inhibited by PP2. Interestingly, the effect of ET-1 on the rate of glucose uptake was abrogated when c-Src was inhibited by PP2 (Figure 7B). Finally, our results show that silencing Cx43 did not up-regulate HIF-1α when the activity of c-Src was inhibited by PP2 (Figure 7C).

## Discussion

Endothelins are involved in several important pathologies of the CNS such as ischemia, gliomas, reactive gliosis and Alzheimer's disease. One of their targets in the CNS is astrocytes, in which endothelins exert a broad range of biological effects. In this sense, the mitogenic effect of ET-1, its ability to inhibit gap junctional communication, to down-regulate Cx43, the protein forming gap junction channels, and to increase the rate of glucose uptake is well known [14], [15], [16], [44]. In previous work we showed that ET-1 increased the rate of glucose uptake in astrocytes by a mechanism that includes the up-regulation of the glucose transporters GLUT-1 and GLUT-3 and the enzymes required to phosphorylate glucose, Hx-1 and Hx-2 [18]. In the present study we sought deeper into the signalling pathway responsible for this process. Our results show that ET-1 promoted an increase in the levels of HIF-1α in astrocytes under normoxic conditions, as it has been previously shown in melanoma and ovarian carcinoma cells [28], [29], [30]. When HIF-1α is silenced in astrocytes, the effects of ET-1 on GLUT-1, GLUT-3, Hx-1, Hx-2 and on the rate of glucose uptake were strongly reduced. Since HIF-1α is a transcription factor for GLUT-1, GLUT-3, Hx-1 and Hx-2 [27], it could be proposed that the effect of ET-1 on the rate of glucose uptake in astrocytes is mediated by HIF-1α.

We have previously shown that the effect of ET-1 on glucose uptake is mediated by the reduction in the expression of Cx43 [26]. Agreeing with this, in this study we show that silencing Cx43 promoted an increase in the levels of HIF-1α and reduced the effect of ET-1 on HIF-1α, indicating that Cx43 participates in the effect of ET-1 on HIF-1α. Cx43 is the main protein forming gap junction channels in astrocytes, which implies that the protein is critical for important functions of astrocytes in the brain [23], [51]. In fact, Cx43 expression can be reduced in response not only to ET-1 but also to various physiological and pathological stimuli (for a review, see [23]). This study contributes to the identification of the signalling pathway evoked after Cx43 down-regulation that results in increased glucose uptake in astrocytes. In this sense, although the relationship between Cx43 and several signalling pathways is well known [34], so far this is the first evidence showing a relationship between Cx43 and HIF-1α.

HIF-1α is mainly regulated by hypoxia but it can also be up-regulated by several mechanisms, including the activity of c-Src [31], [32], [33], a non-receptor tyrosine kinase involved in the regulation of cell proliferation. Interestingly, the intracellular carboxyl tail of Cx43 interacts with c-Src [40], [41], [42], and this interaction modulates reciprocally their activities. Thus, phosphorylation of Cx43 by c-Src reduces gap junctional communication [40], [52], [53] while the increase in Cx43 levels inhibits c-Src activity, at least in rat glioma cells [43]. Agreeing with this, our results show that both the treatment with ET-1 and with Cx43-siRNA increased c-Src activity in astrocytes. In addition, when c-Src activity was inhibited neither ET-1 nor silencing Cx43 were able to up-regulate HIF-1α. Consequently, the effect of ET-1 on glucose uptake was abrogated when c-Src is inhibited. It is well documented that the inhibition of gap junctional communication in astrocytes promoted by ET-1 and other gap junction uncouplers increases proliferation and glucose uptake [16], [18], [24], [26], however the molecular mechanism linking these cellular events is unknown. In this study we provide evidence that c-Src is activated by ET-1 and by silencing Cx43. c-Src is a well known regulator of cell proliferation [54] and this study and the work carried out by other laboratories [31], [32], [33] show that c-Src can activate HIF-1α and consequently glucose uptake. Therefore, it could be proposed that c-Src could be the mediator that links the increase in cell proliferation and glucose uptake found in astrocytes after inhibiting gap junctional communication or reducing Cx43 expression.

Furthermore, our results confirm that the level of Cx43 expression is important to regulate c-Src activity. Thus, the up-regulation of Cx43 in glioma cells reduces the high c-Src activity found in these cells [43], while in this study we show that silencing Cx43 activates c-Src in astrocytes. It should be mentioned that this effect could be due to the absence of Cx43 function (gap junctional communication) or to the absence of Cx43 interaction with other proteins, such as c-Src. Changes in Cx43 expression are accompanied by changes in cell proliferation, thus restoration of Cx43 in glioma cells decreases the rate of proliferation [43], [51], [55], [56], while silencing Cx43 increases the rate of astrocyte proliferation [26]. Consequently, it could be proposed that the interaction between Cx43 and c-Src could be an important step in the regulation of cellular events, such as cell proliferation. In this context, the interaction between Cx43 and c-Src has been proposed to be responsible for the regulation of P2Y1 purinergic receptors, which are involved in glial calcium signal transmission and in the migration of neural progenitor cells [57]. Together, these data suggest a relevant role of the interaction between Cx43 and c-Src in the CNS.

**Figure 8:**
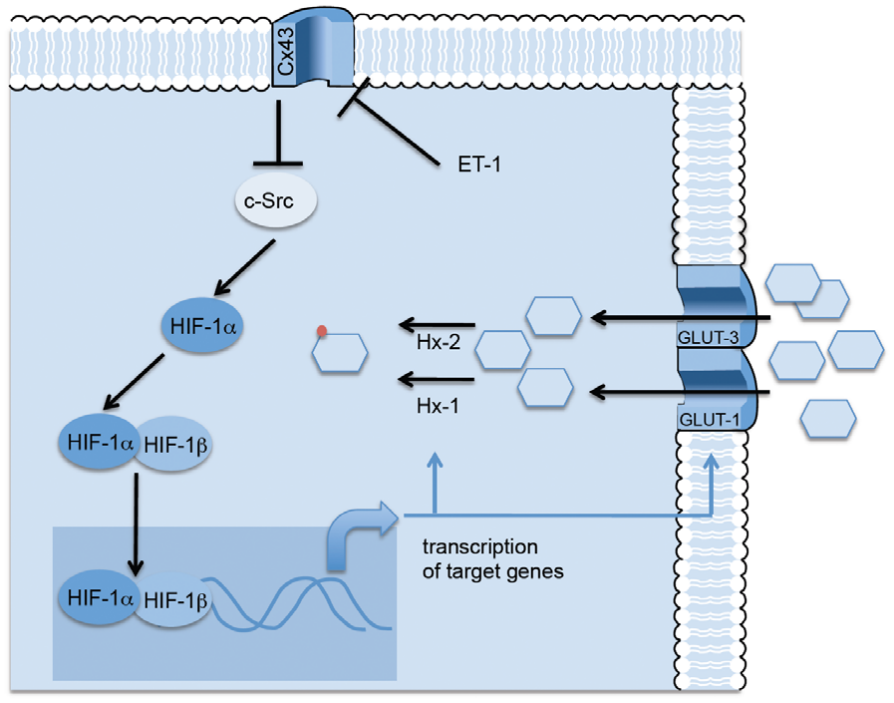
Proposed mechanism. Since Cx43 inhibits c-Src activity [43], it could be proposed that ET-1 by reducing Cx43 activates c-Src, which in turn increases HIF-1α. HIF-1α dimerizes with HIF-1ß and translocates to the nucleus. The transcriptional activity of HIF induces the synthesis of the machinery required to augment the rate of glucose uptake in astrocytes, i.e., the glucose transporters, GLUT-1 and GLUT-3 and the enzymes required for glucose phosphorylation, type I and type II hexokinase (Hx-1 and Hx-2).

As mentioned above, endothelins are involved in several pathologies of the CNS such as gliomas [11]. It should be mentioned that reduced expression of Cx43 [58], [59], [60], [61], high c-Src activity [62] and activation of the HIF-1 pathway [63] are all common features of gliomas. In addition, glioma cells, like many other cancer cells adapt their metabolism to the tumour environment, a process known as “Warburg Effect” that begins by an increase in the rate of glucose uptake and a metabolic shift to aerobic glycolysis, which is associated with a survival advantage as well as the generation of substrates necessary in rapidly proliferating cells [64]. For instance, enzymes such as Hx-2 are key mediators of aerobic glycolisis and promote tumour growth in gliomas [65]. In this study, we suggest that these events could be linked in a common pathway. Thus, our results suggest that ET-1 by down-regulating Cx43 activates c-Src, which in turn up-regulates HIF-1α leading to the transcription of the machinery required to increase the rate of glucose uptake in astrocytes (Figure 8). These metabolic changes are probably designed to sustain the higher rate of cell proliferation observed under these circumstances [66]. ET-1 participates in the progression of different tumours [67], however, whether the presence of ET-1 in gliomas [11] activates the pathway reported in this study (Figure 8) to promote the growth and progression of these tumours remains to be elucidated and warrant further exploration.

## Materials and Methods

### Materials

Dulbecco's modified Eagle medium (DMEM), penicillin, streptomycin, poly-L-lysine, ET-1, and protease inhibitors were obtained from Sigma-Aldrich Chemical Co. (Madrid, Spain). Fetal calf serum (FCS), DNase, bovine serum albumin and trypsin were from Boehringer Mannheim (Barcelona, Spain). Optimen and Lipofectamine 2000 were purchased from Invitrogen (Barcelona, Spain). Monoclonal antibody against glyceraldehyde-3-phosphate dehydrogenase (GAPDH) and small interfering RNAs (siRNAs) were obtained from Genelink (New York, USA). 2-Deoxy-D[1-^14^C]glucose was from Amersham Pharmacia Biotech (Barcelona, Spain). The c-Src inhibitor, PP2 and the control PP3 were obtained from Calbiochem (Nottingham, United Kingdom). Mouse monoclonal antibody against Cx43 (610062) was from Transduction Laboratories, Inc. (BD Bioscience Pharmigen, San Diego, CA, U.S.A.). Mouse monoclonal antibody against Hx-1 (MAB1532) and Hx-2 (MAB1629) and rabbit polyclonal antibody against GLUT-1 (AB1340) and GLUT-3 (AB1344) were from Chemicon International Inc (Madrid, Spain). Rabbit polyclonal antibody against total c-Src (2108) and rabbit polyclonal antibody against Y416 c-Src (2101) were from Cell Signaling Technology (Boston, EEUU). Rabbit polyclonal antibody against HIF-1α (NB100-479) was from Novus Biologicals (Colorado, EEUU). Polyvinylidene fluoride membranes (PVDF) were from Millipore Corporation (Bedford, U.S.A.). The Bio-Rad protein assay and polyacrylamide were from Bio-Rad (Madrid, Spain). X-ray films were from Fujifilm (Madrid, Spain). Other chemicals were purchased from Sigma-Aldrich Chemical Co. (Madrid, Spain) or Merck (Barcelona, Spain).

### Animals

Albino Wistar rats, fed ad libitum on a stock laboratory diet (49.8% carbohydrates, 23.5% protein, 3.7% fat, 5.5% wt/vol minerals and added vitamins and amino acids), were used for the experiments. Rats were maintained on a 12-h light–dark cycle. Postnatal day 1 newborn rats were used to prepare astrocyte cultures. The animals were obtained from the animal facility of the University of Salamanca and their use for this study was approved by the bioethics committee of this institution.

### Cell cultures

Astrocytes in primary culture were prepared from the forebrains of 1- to 2-day-old Wistar rats as previously described [68]. Briefly, animals were decapitated and their brains immediately excised. After removing the meninges and blood vessels, the forebrains were placed in Earle's balanced solution containing 20 mg/mL DNase and 0.3% (w/v) bovine serum albumin. The tissue was minced, washed, centrifuged and incubated in 0.025% (w/v) trypsin and 60 mg/mL DNase for 15 min at 37°C in a shaking water bath. Trypsinization was completed by adding DMEM containing 10% (v/v) FCS. The tissue was then dissociated by trituration, passing it eight times through a siliconized Pasteur pipette, and the supernatant cell suspension was recovered. This procedure was repeated and the resulting cell suspension was centrifuged. The culture medium was DMEM (with 5 mM glucose) supplemented with 10% (v/v) FCS, penicillin (50 U/mL) and streptomycin (50 mg/mL). The cell pellet was resuspended in a known volume of culture medium and plated onto poly-L-lysine-coated Petri dishes or flasks at a density of 1.5×10^5^ cells/cm^2^. Cells were incubated at 37°C in an atmosphere of 95% air/5% CO_2_ with 90–95% humidity. After 3 days, cytosine arabinoside (10 mM) was added to the culture medium for 2 days to prevent the growth of microglia and cells from the O-2 lineage. The culture medium was renewed with a fresh one twice a week. Under our experimental conditions, 90–95% of the cells were astrocytes as determined by immunostaining against glial fibrillary acidic protein [69], [70], [71]. Experiments were carried out after 21 days in culture.

### Cell treatments

The compounds tested were 0.1 µM ET-1, 100 ng/mL PP3 or 100 ng/mL PP2. PP2 and PP3 were preincubated for 1 hour and were present in the solutions used throughout the experiments. Incubations were performed for the indicated times in culture medium at 37°C in an atmosphere of 95% air/5% CO_2_ with 90–95% humidity.

### Transfection of siRNA

Conditions were similar to those reported previously [26], [43]. Cells were transfected with the double-strand siRNA (50 nM) complexed with 2.5 µl/ml Lipofectamine 2000 in culture medium without antibiotics. The sequences of siRNAs were as follow: Cx43-siRNA: sense 5′-gcugguuacuggugacagatt-3′ and antisense 5′-ucugucaccaguaaccagctt-3′; HIF-1α siRNA: 5′-cugauaacgugaacaaauatt-3′ and antisense 5′-uauuuguucacguuaucagtt-3′ and a validated NT-siRNA provided by Ambion used as a negative control. Other siRNA sequences for HIF-1α were tested (sense 5′-cauugaagaugaaaugaaatt-3′, antisense 5′-uuucauuucaucuucaaugtt-3′ and sense 5′-cuguugaucuuauaaugautt-3′, antisense 5′-aucauuauaagaucaacagtt-3′) and the same phenotype was observed (Figure S1). The cells were maintained in the presence of the oligonucleotides in culture medium without antibiotics and after 6 h, the medium was replaced with DMEM plus 10% FCS with antibiotics. The extent of siRNA-mediated down-regulation of protein expression was evaluated in Western blots. Cell treatments were performed 48 hours after siRNA transfections.

### 2-Deoxyglucose uptake

Conditions were similar to those reported previously [17], [18], [26]. After the indicated treatments, cells were incubated with DMEM containing 2-deoxy-D[1-^14^C]glucose (750 dpm/pmol) for 30 min. Then, cells were washed with ice-cold PBS and were lysed by adding 500 µL of 10 mM NaOH containing 0.1% Triton X-100. An aliquot was assayed for [^14^C] by liquid scintillation counting (efficiency 95%) and another aliquot was used to measure protein concentration.

### Western blotting

Twenty-four hours after the treatments, proteins were extracted from the cells using 2% sodium dodecyl sulphate (SDS) in 5 mM Tris–HCl, pH 6.8, containing 2 mM EGTA, 2 mM EDTA, 2 mM phenylmethylsulphonyl fluoride, 0.5 µg/mL antipain, 0.5 µg/mL pepstatin, 0.5 µg/mL amastatin, 0.5 µg/mL leupeptin, 0.5 µg/mL bestatin, 0.5 µg/mL of trypsin inhibitor, sodium fluoride (NaF) 1 mM and sodium orthovanadate (Na_3_VO_4_) 100 µM. The protein extract (80 µg) was applied to a 10% SDS–Polyacrilamide gel under reducing conditions and then transferred to a PVDF membrane. The membranes were cut into several strips to be immunoblotted with distinct antibodies, thus allowing for comparative analysis of the amount of each protein in the same sample. Membranes were then blocked with 10% fat-free dried milk in TTBS and then exposed to primary antibody against Cx43 (1∶100), Hx-1 (1∶500), Hx-2 (1∶500), GLUT-1 (1∶500), GLUT-3 (1∶500), c-Src (1∶500), Y416 c-Src (1∶500) or HIF-1α (1∶500), for at least 4 h. Mouse antibody against GAPDH (1∶5000) was used as a loading control. Peroxidase-conjugated anti-rabbit IgG or peroxidase conjugated anti-mouse IgG were used and developed with a chemiluminiscent substrate. Membranes were exposed to X-ray films. When necessary, blots were stripped and re-probed with other antibodies. X-ray films were scanned and densitometry analysis of the bands was performed using image-analyzer software (SCION IMAGE, based on NIH Image, Wayne Rasband, National Institutes of Health, Bethesda, MD, USA). The amounts of GAPDH recovered in each sample served as loading control and the values for each protein were normalized to their corresponding GAPDH level. The results are expressed as percentages of the values found in the controls.

### Protein Determinations

Protein concentrations were determined by the Bradford method [72], using bovine serum albumin (BSA) as the standard.

### Statistical analyses

The results are the means ± S.E.M. of at least three independent experiments. Statistical analyses were carried out with Student's *t*-test or One-Way ANOVA followed by Tukey test when comparing more than two variables. Values were considered significant when p<0.05.

## Supporting Information

Figure S1 **Silencing HIF-1α in astrocytes by siRNA.** Astrocytes were transfected with NT-siRNA or with 3 different sequences of siRNA specific for HIF-1α (sequence 1: sense 5′-cauugaagaugaaaugaaatt-3′, antisense 5′-uuucauuucaucuucaaugtt-3′; sequence 2: sense 5′-cugauaacgugaacaaauatt-3′ and antisense 5′-uauuuguucacguuaucagtt-3′; sequence 3: sense 5′-cuguugaucuuauaaugautt-3′ and antisense 5′-aucauuauaagaucaacagtt-3′). At the indicated times the proteins were extracted and HIF-1α levels were analysed by Western blot. The results are expressed as percentages relative to the level found in cells transfected with NT-siRNA. ***p<0.001 versus NT-siRNA. Sequence 2 was selected for the following experiments because it showed the higher down-regulation after 48 h and the reduction was maintained for 72 h.
(TIF)
