# Supplementary figures and images for: HIF-1 and c-Src Mediate Increased Glucose Uptake Induced by Endothelin-1 and Connexin43 in Astrocytes
Source: PLoS One. 2012 Feb 23;7(2):e32448. doi: 10.1371/journal.pone.0032448 (PMC3285680; doi:10.1371/journal.pone.0032448)

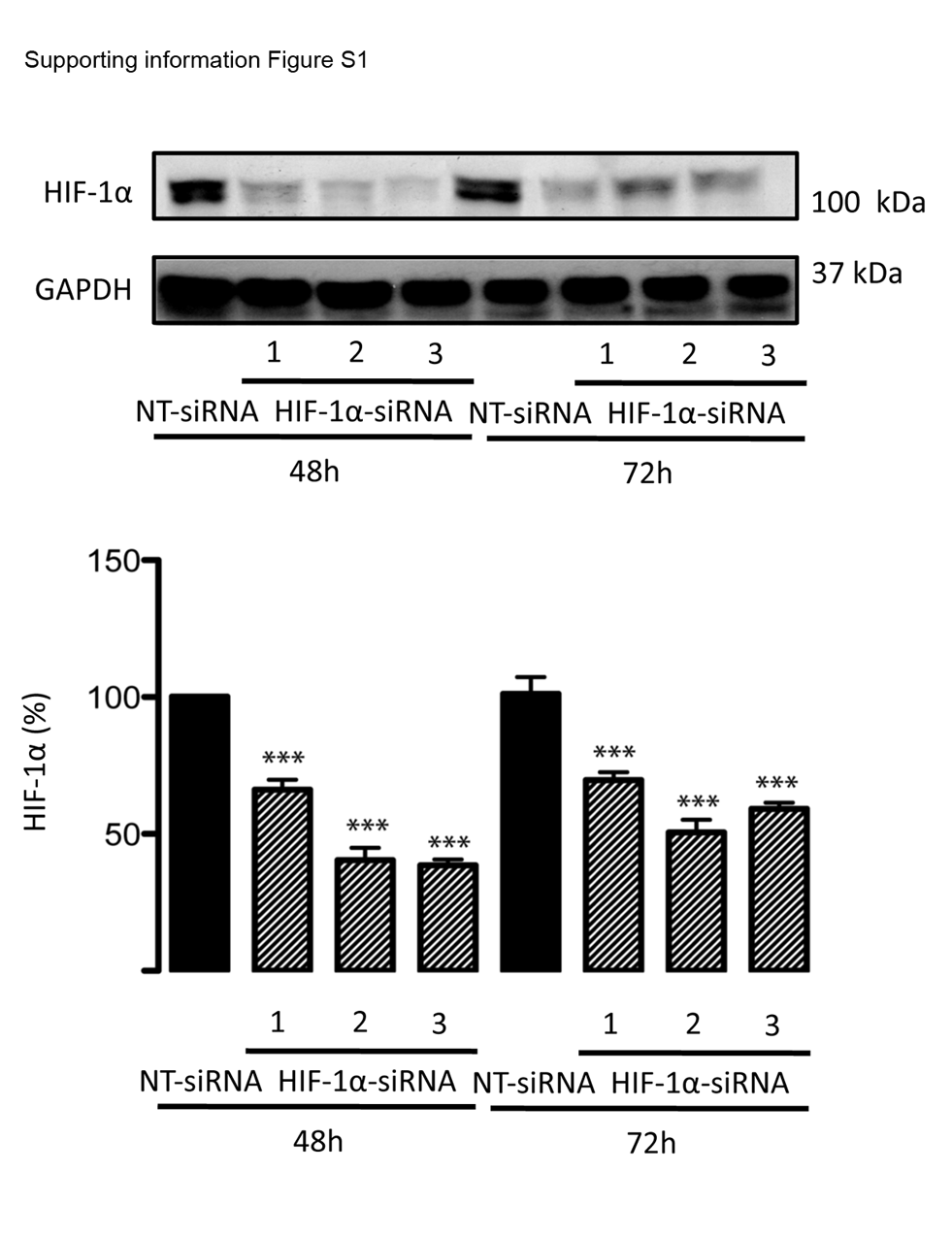

Supplement: Figure S1 — Silencing HIF-1α in astrocytes by siRNA. Astrocytes were transfected with NT-siRNA or with 3 different sequences of siRNA specific for HIF-1α (sequence 1: sense 5′-cauugaagaugaaaugaaatt-3′, antisense 5′-uuucauuucaucuucaaugtt-3′; sequence 2: sense 5′-cugauaacgugaacaaauatt-3′ and antisense 5′-uauuuguucacguuaucagtt-3′; sequence 3: sense 5′-cuguugaucuuauaaugautt-3′ and antisense 5′-aucauuauaagaucaacagtt-3′). At the indicated times the proteins were extracted and HIF-1α levels were analysed by Western blot. The results are expressed as percentages relative to the level found in cells transfected with NT-siRNA. ***p<0.001 versus NT-siRNA. Sequence 2 was selected for the following experiments because it showed the higher down-regulation after 48 h and the reduction was maintained for 72 h. (TIF) [file pone.0032448.s001.tif]
